# Childhood-Onset Systemic Lupus Erythematosus: Southeast Asian Perspectives

**DOI:** 10.3390/jcm10040559

**Published:** 2021-02-03

**Authors:** Swee Ping Tang, Sern Chin Lim, Thaschawee Arkachaisri

**Affiliations:** 1 Pediatric Rheumatology Unit, Selayang Hospital, Batu Caves 68100, Malaysia; tangsweeping@gmail.com; 2 Department of Pediatrics, Universiti Teknologi MARA (UiTM), Sungai Buloh 47000, Malaysia; sernchin@gmail.com; 3 Rheumatology and Immunology Service, Department of Pediatric Subspecialties, KK Women’s and Children’s Hospital, 100 Bukit Timah Road, Singapore 229899, Singapore; 4 Pediatric Academic Clinical Program, Duke-NUS Medical School, 8 College Road, Singapore 169857, Singapore

**Keywords:** child, lupus, Southeast Asia, clinical features, disease activity, disease damage

## Abstract

Childhood onset systemic lupus erythematosus is a rare disease that is more common amongst Southeast Asian children compared to the West. It is typified by a peripubertal onset and a female preponderance, which increases with advancing age. Organs commonly involved at diagnosis include haematological, renal, and mucocutaneous. Fever, malar rash, and cutaneous vasculitis are common. Lupus nephritis is typically proliferative especially Class IV and contributes to both disease activity and damage. Antinuclear antibody and anti-dsDNA positivity are both prevalent in this region. Disease activity is higher than Western cohorts at onset but responds to therapy reducing to low disease activity by six months. However, organ damage occurs early and continues to accumulate over the time, a consequence of both active disease (neurological and renal systems) and steroid-related complications especially in the eye (cataract and glaucoma) and musculoskeletal systems (avascular necrosis). Infections remain the leading cause of death and mortality in this region is highly variable contributed by the heterogeneity in social economic status, healthcare access, and availability of paediatric rheumatology expertise in the region.

## 1. Introduction

Systemic lupus erythematosus (SLE) is a prototypic chronic multisystem autoimmune disease that is highly heterogenous in its clinical manifestations and severity. Although SLE predominantly affects young women, children and adolescents contribute to 15–20% of this population [1,2]. Adults and children with SLE share many similarities in terms of diagnosis and treatment, nevertheless there are important differences. Childhood-onset SLE (cSLE) often presents acutely and runs an aggressive course affecting major organs in the body, especially the kidneys, the brain, and blood systems [3,4,5]. When compared to adult-onset SLE (aSLE), children have higher disease activity and disease burden, resulting in more long-term disease damage, morbidity, and mortality [1,3,5,6,7].

In addition to these differences between adult and childhood-onset SLE, genetics and ethnicity have also been reported to influence SLE disease expression contributing to clinical heterogeneity. Both Asian children and adults with SLE are prone to more serious clinical manifestations and have a poorer outcome when compared to white populations [8,9,10,11,12,13,14].

Southeast Asia (SEA) comprises of 11 countries of diverse history, religion, and culture but bounded by geographical location. They include Brunei, Cambodia, Timor-Leste, Indonesia, Laos, Malaysia, Myanmar, Philippines, Singapore, Thailand, and Vietnam. Of these, only Brunei and Singapore are developed countries with high income, whilst the remainder are middle- or low- income countries, with significant variation in access to healthcare.

In this paper, we aim to review and summarize the epidemiology and demographics, clinical manifestations, laboratory features, and outcomes of childhood-onset systemic lupus erythematosus in Southeast Asia. Published papers regarding the aforementioned information on childhood-onset systemic lupus erythematosus from SEA were obtained, analyzed, and then compared to the corresponding information reported elsewhere.

## 2. Epidemiology and Demographics

SLE is a rare childhood disorder with an estimated incidence of 0.3–0.9 per 100,000 children-years and a prevalence of 3.3–8.8 per 100,000 children [15]. Arkachaisri estimated the cSLE prevalence in Singapore to be 14.2 per 100,000 children, which is higher than published data including that of other Asian countries like Taiwan with a prevalence of only 6.3 per 100,000 [16,17]. This could be due to differences in ethnic composition, study methodology including ages of inclusion as well as varying referral practices in each country. However, the true incidence and prevalence in this region is still unknown due to the absence of nationwide population-based epidemiological studies. Pediatric rheumatology services are still significantly lacking in SEA with only 48 pediatric rheumatologists, of which 45 are concentrated in 4 countries, 3 countries with a single pediatric rheumatologist each and 4 countries with none [16,18]. Issues of under reporting and under diagnosis of SLE, especially in the very young, may be prevalent due to the lack of pediatric rheumatology expertise [17,19]. In addition, published studies in this region are all from single centers limiting generalizability and reflecting referral bias.

The onset of cSLE is reported to peak during the peri-pubertal years with a median age of onset between 11–12 years and the disease is rare under the age of 5 years [20]. In SEA, (Table 1) most patients are diagnosed between the ages of 11–13 years, which is comparable with other multiethnic cohorts in the West. Thailand (mean 9.7 years) and Malaysia (median 10.8 years) have a slightly younger age at SLE diagnosis potentially reflecting a shared genetic pool as well as environmental influence due to their regional proximity [21,22]. In addition, the lower mean age at diagnosis in Thailand is partly attributed to the younger population of study defined by an upper age limit of 16 years instead of 18 years. In another study conducted in southern Thailand, the mean age at diagnosis (11.6 ± 2.6 years) was more reflective of the region [23].

On the other hand, Gulay and Dans reported a higher age of diagnosis in the Philippines (mean 14.7 ± 2.7 years), but this could be due to a skewed population as their youngest patient was 8 years of age and the peak for the predominantly female cohort was 17 years [24]. Another study in the Philippines reported a mean age of diagnosis of 12.3 years ± 2.9, more in keeping with other published data and supporting this postulation [25]. Dung et al. also did not report any cSLE under the age of 5 years in Vietnam but this may be due to underdiagnosis as the very young lupus normally presents with non-specific symptomatology [19,26]. 

The female preponderance observed in cSLE with a typical female to male ratio of 5:1 is similarly seen in this region [13,17,27,28]. Philippines has a higher female preponderance (10:1) but this could be attributed to an older cohort [24]. Interestingly, Malaysia also reports a higher female preponderance of 7.3: 1 although their cohort is younger than the average reported in this region and this may reflect a true female preponderance [21]. Two countries in this region also reported on age-related gender variations comparable to published reports whereby the female preponderance increases with increasing age [1,26,28]. Lim et al. in Malaysia reported female: male ratios of 2.3:1 in 0–5 years, 8.3:1 in 6–12 years, and 15:1 in 13–18 years, whilst Supavekin et al. in Thailand reported female preponderance ratios of 2:1 in <5 years, 5:1 for 5–9.9 years, and 7.9:1 in 10–14.9 years [21,22]. The overall female preponderance magnified by the onset of puberty emphasizes the importance of hormonal influence in disease expression.

Tan et al. also found that the Malay ethnicity was disproportionately over-represented in the multi-ethnic Singapore population, suggesting possible genetic influence and a racial predilection, but this was not similarly seen in Malaysia, a predominantly Malay population [21,29].

## 3. Clinical and Laboratory Features

The most common organ systems involved at diagnosis in SEA are the hematological, renal, and mucocutaneous systems [30]. The involvement of major organs at diagnosis is consistent with published data from other pediatric series and supports the findings that cSLE tends to be more severe compared to aSLE [1,3,5,8,32,33]. 

Hematological abnormalities are very common in SEA with almost all (98.4%) patients in Singapore being affected [29]. Tan et al. in his study reported that majority (81.3%) had lymphopenia at presentation suggesting its utility when considering a differential diagnosis of SLE in children. Hemolytic anemia was also common in most SEA countries (22–34.6%) and comparable with other published pediatric cohorts although thrombocytopenia featured more prominently in the Malaysian (41.8%) and Singaporean (32.8%) cohorts [21,29]. 

Arthritis was present in high numbers in Singapore (56.3%) and Vietnam (58%), which is comparable to Western pediatric studies (61–65%) but still less than in adult studies [8,19,26,29]. The other countries in the region reported less arthritis, especially the Philippines (21.7%) and Thailand (31.9%) [22,24]. There is a possibility that the presence of other more severe organ involvement like renal or central nervous system (CNS) detracts attention from the musculoskeletal presentations which may be mild or asymptomatic.

Malar rash is the predominant cutaneous manifestation in this region with a high prevalence affecting 1/2 to 2/3 of the cohorts, which is comparable to studies from the West [11,32,34]. Singapore has the lowest prevalence of 45.3% in SEA but even then, malar rash remains the commonest type of cutaneous manifestation [29]. On the contrary, discoid rash is uncommon in this region (2.0–15.6%) comparable to other cSLE studies, except for the Philippines where it was present in up to 1/3 of their cohort [21,22,24,29]. The low prevalence of discoid rash in the region is not at all surprising given that it is more common in African Americans [35]. Other common mucocutaneous manifestations include oral ulcers seen in about 1/3 of the patients in this region, except for a surprisingly higher prevalence of up to 50% in Malaysians and Filipinos [21,24]. There is also a high prevalence of photosensitivity amongst the Filipinos and Vietnamese which is not seen in the other countries in this tropical region [19,24].

Renal disease is a main contributor to long-term morbidity and mortality in lupus and is more common in children when compared to adults [1]. It is also more common in Asians compared to Caucasians [36]. Therefore, it is not surprising that lupus nephritis featured prominently in this region as one of the main organs involved at diagnosis. In Thailand and Vietnam, about 8 in every 10 newly diagnosed children with lupus have renal disease [19,22]. Lim et al. and Tan et al. reported the lowest rates of renal disease at diagnosis in this region, 39.7% in Malaysia and 40.6% in Singapore, which are more comparable with Western studies [11,21,28,29,31]. Tan et al. believes that the low rates accurately reflect the Singaporean population due to their unique universal catchment of children with rheumatic diseases [29]. Lim et al. however felt that the low rates in Malaysia were contributed by the population studied as some children with lupus nephritis may have been solely treated by paediatric nephrologists, suggesting that the prevalence of renal disease may be truly higher amongst Asian children [21,29].

The predominant histopathology of lupus nephritis (LN) in Asian children was Class IV lupus nephritis (39.4–54%), which is similarly reflected in other Western countries like USA (38%) and Canada (46%) [11,17]. Class IV LN is also the commonest histopathological type in SEA and together, the proliferative types of LN (Class III and IV) accounted for 64.3–91.6% of those with biopsy proven lupus nephritis (Table 2). A proliferative pattern translates to higher disease activity contributing to the increased burden of cSLE in this region. There was absence of other milder classes of lupus nephritis in Vietnam and Thailand, but this probably reflects the local practice where biopsies were only performed on patients with significant renal presentations [19,22]. Although less than half of the LN children in Philippines had renal biopsies performed due to financial constraints, the predominant histology was still Class IV and interestingly, there was a high percentage of Class II as well [24].

At diagnosis, neurological diseases were generally uncommon in this region, ranging from 14.1–20%, which is similar to Western literature [19,21,22,29,31]. An exception to this is in the Philippines, where Gulay and Dans reported neurological diseases in up to almost 1/3 of their cohort [24]. This is a surprising finding given that neurological disease is more common in the young cSLE but the Philippine cohort comprised older children [26]. Tan et al. reported a low prevalence of 14.1% in Singapore, which was due their stricter definition of neurologic disorders including only manifestations of seizures and psychosis whilst other studies in the region had included manifestations like headache and other more subtle cognitive or mood disorders that contributed to a slightly higher prevalence [21,22,24,37,38]. The overall extent of neuropsychiatric involvement could be higher than reported as disorders such as cognitive and mood disorders are frequently not diagnosed and not included in many studies. 

Serositis is comparable to other reports in the world, except for a higher occurrence in Philippines (26.9%) and Vietnam (36%) [19,24]. The low prevalence of serositis could be because mild and asymptomatic patients may not have been routinely investigated in some patients.

Constitutional symptoms, especially fever, malar rash, and cutaneous vasculitis, are known to be more common in children as evident in our regional SEA cohorts [21,24,29,32,39]. 

Antinuclear antibody (ANA) positivity was generally high in this region with low incidence of ANA negative lupus (<4%). Dung et al. reported a low ANA positivity of 67% in Vietnam but interestingly had the highest anti-dsDNA antibodies in the region and high prevalence of renal disease [19,28,31]. Hoffman et al. found that anti-dsDNA antibodies were positively associated with glomerulonephritis and urinary casts in cSLE. [39] However, there was no such association seen in the only study in the region which looked specifically into this [29].

## 4. Disease Activity, Disease Damage, Long-Term Outcomes, and Mortality

Children with SLE have significantly more active disease at presentation as measured by SLE Disease Activity Index (SLEDAI) scores when compared to adults [3,11,28,40]. This difference is due to the higher prevalence of renal and neurological disease in children, both of which are common in this region [11,41].

SLEDAI scores at diagnosis in SEA (Table 3) indicate high disease activity (16.7–23.8) and are higher compared to other published Western cohorts [11,19,21,28,29]. Dung et al. reported the highest SLEDAI scores at diagnosis in this region, contributed to by the high prevalence (82%) of renal disease and high proportion (85%) of the proliferative types [19]. However, despite high disease activity scores at diagnosis, good treatment response can be observed six months after initiation of treatment, which is similarly seen in Western cohorts [11,21,28,29]. Lim et al. also reported a tendency towards slightly higher disease activity in those under the age of five years but with no significant gender difference [21].

Along with higher disease activity, children with SLE also exhibit more damage when compared to adults [3]. Disease damage can be the result of high disease activity, often associated with major organ involvement, which is independent of the patient’s ethnicity, or due to complications of treatment [21,42]. There are limited reports of disease damage as measured by SLICC/ACR Damage index (SDI) in this region. Lim et al. reported a high incidence of damage present as early as one year after diagnosis in 31.9% of patients (SDI 0.4). The damage accrual increased to 50.0% (SDI 0.90) at 5 years and 60.0% (SDI 1.9) at 10 years follow-up. There was no significant difference in the SDI amongst the various ethnicities in the Malaysian study but the numbers of the minority ethnicities were too small to draw a proper conclusion. Although the rates of damage are high in this Malaysian cohort, it is still comparable to the 5-year SDI of 56.1% reported by Brunner et al. in a Canadian cohort [8,21]. However, interestingly, the 5-year SDI in Singapore is very low (12.6%) and postulated to be due to the lower prevalence of renal and neurological diseases compared to other countries in this region as well as easy healthcare accessibility and judicious use of immunosuppressive medications [29].

There is paucity of renal survival data in SEA with end stage renal failure rates reported between 3.14% in Malaysia and 6.6% in Thailand, which are high compared to 0.5% in the United Kingdom cohort [21,22,23,28]. Similarly, neurological damage especially seizures were also common (19–22%) and higher than the 8% reported by Watson et al. [21,24,28]. Steroid related complications were present with ocular complications (cataracts and glaucoma) in 1.2–29.0% and avascular necrosis in 5.6–8.7% [21,24,29,43]. This is not unexpected as cSLE generally requires more steroids compared to aSLE and with improved survival, the effects of prolonged steroid therapy become manifested [43]. Growth complications are not assessed by the SDI but are integral in the management of a child with lupus. Lim et al. reported very high rates of growth retardation (38.2%) amongst the Malaysian children when compared with other cSLE cohorts in SEA (5.1% in Philippines) or with the rest of the world (10–28.3%) [8,21,24,27,28]. This could be attributed to differences in the definitions of growth failure as well as the high disease activity seen in our Asian population requiring prolonged steroid therapy despite additional immunosuppressive medications. There were also no permanent premature gonadal failure and no malignancies reported but most of the regional cohorts do not have long enough follow-up to accurately generalise this data [21].

Although the survival of children with SLE has improved over the years to more than 90% since the 1980s, the mortality rates in children are still higher when compared to adults [15,34,44]. In addition, there are striking differences in 10-year paediatric survival rates between high income countries (HIC), 0.97 versus low/middle income countries (LMIC), 0.79 [45]. Singapore, one of the two HIC in SEA, had previously reported high mortality of 25% in their 10-year cSLE cohort back in 1987 [46]. However, a more recent study showed marked improvement in survival with the country’s economic development and accessibility to healthcare with a mortality of only 1.6% in a 5-year cohort on par with other developed countries in the world [29,47,48]. Mortality rates in Thailand (23.3–31.2%) remain high despite improvement over the years with most deaths occurring within the first year of presentation [49,50]. Dung et al. reported similar findings of high incidence of early deaths in 8.9% of their cohort in Vietnam within the first six months of diagnosis [19]. These could reflect the high prevalence of renal disease in these countries with poor healthcare accessibility, delayed diagnosis, and limited paediatric rheumatology expertise [45,49]. Meanwhile, the mortality rates of 7.6% (5-year follow-up) in the Philippines and 5.7% (10 year follow up) in Malaysia are comparable to other developed countries [21,24]. The leading cause of death for cSLE in this region is infections in contrast to SLE and its related complications reported in Canada and Latin America [11,19,21,22,24,29,34]. Probable explanations again point to the low socio-economic status of most countries in this region coupled with poor health education, awareness, and delayed presentations [19,22,24].

With the improved survival of children with SLE in this region, majority will today live until adulthood. The concept of planned transition from pediatric to adult rheumatology care thus becomes more important. There are currently no published papers on transitional care in SEA and only two countries (Malaysia and Singapore) have formal transitional care programs [18,51]. This is not surprising given that many countries in this region are still struggling with a lack of pediatric rheumatologists. Nevertheless, transitional care development should not be neglected but encouraged to be developed in tandem with pediatric rheumatology service development in the region.

## 5. Conclusions

SLE in Southeast Asian children share many similar features with other cSLE cohorts around the world. Unique characteristics include a higher disease activity at presentation and the predominance of proliferative renal disease. Disease damage occurs early from both active disease and steroid-related complications. Mortality is variable in the region affected by heterogeneity of healthcare access and socio-economic status of the region. Knowledge gaps on cSLE still abound in SEA and calls for more collaborative research to improve the wellbeing and outcomes of Southeast Asian children with lupus.

## Figures and Tables

**Table 1:** Clinical and laboratory features of childhood-onset SLE at diagnosis (SLICC criteria) [30].

	Characteristics	Tan et al. (2015) [29] *n* = 64	Lim et al. (2020) [21] *n* = 141	Gulay and Dans (2011) [24] *n* = 78	Dung et al. (2012) [19] *n* = 45	Supavekin et al. (2005) [22] *n* = 10	Kim et al. (2019) [31] *n* = 276	Hiraki et al. (2008) [11] *n* = 256	Watson et al. (2012) [28] *n* = 198
	Country	Singapore	Malaysia	Philippines	Vietnam	Thailand	Canada	Canada	UK
	Multicentre/Single centre	Single	Single	Single	Single	Single	Multi-center	Single center	Multi-center
	Ethnicity	Chinese 45.3%, Malays 28.1%, Indians 9.4%,	Malay 61.7% Chinese 8.4% Indians 6.4%	Filipino	Vietnamese	Thai	Asian 40.2% Caucasian 36.6% Blacks 10%	Mixed, predominant Caucasian	Mixed, predominant Caucasian 52%
	Gender (F:M)	5:1	7.3:1	10:1	4:1	6.2:1	5.1:1	4.7:1	5.6:1
	Age at diagnosis (years, mean ± SD or median, IQR)	11.9 ± 2.6	10.8 (IQR 9.0–12.0)	14 ± 2.7	12.8 ± 2.5	9.7 + 2.8	12.7 + 3.3	13.3 + 3.2	12.6 (IQR 10.4–14.5)
**Clinical and laboratory features (%)**	Acute cutaneous lupus Malar rash Photosensitivity	45.3 15.6	56.0 NA NA	65.3 55.1	67.0 53.0	53.5 21.8	63.0 33.5	61 17	59 30
	Chronic cutaneous lupus	15.6	15.6	32.0	13.0	2.0	3.9	NA	10
	Oral ulcers	32.8	48.9	53.8	38.0	31.7	31.1	12	40
	Nonscarring alopecia	31.3	41.8	39.7	NA	13.9	NA	22	NA
	Synovitis	56.3	42.6	21.7	58.0	31.7	61.1	61	65
	Serositis	15.6	18.4	26.9	36.0	12.9	19.5	13	20
	Haemolytic anaemia	28.1	22.0	8.9	NA	34.6	23.0	23	27
	Thrombocytopenia	32.8	41.8	14.1	NA	13.9	29.6	29	20
	Leukopenia or Lymphopenia	67.2	51.1	21.7	NA	NA	41.6	29	73
	Renal Disorder	40.6	39.7	62.8	82.0	86.2	33.9	45	35
	Neurologic Disorder	14.1	16.3	30.7	16.0	20.8	12.8	16	9
**Immunological** **features (%)**	Anti-Nuclear Antibodies	98.4	96.5	98.5	67.0	96.0	95.7	NA	84
	Anti-ds-DNA antibodies	90.6	74.5	23.0	95.0	65.3	66.2	72	69
	Anti-smith antibodies	37.5	35.5	NA	NA	29.7	29.6	34	22
	Anti-phospholipid antibodies	39.1	14.9	NA	NA	NA	42.8	32	36
	Low complement	NA	92.2	8.9	NA	80.5	NA	NA	NA
	Direct Coombs test	NA	64.5	8.9	83.0	34.6	NA	23	NA

SLE, systemic lupus erythematosus; NA: not available; SD: standard deviation; IQR: interquartile range.

**Table 2:** Histopathological classification of childhood-onset lupus nephritis in Southeast Asia.

	Tan et al. [29] *n* = 64	Lim et al. [21] *n* = 141	Vachvanichsanong et al. [23] *n* = 78	Gulay and Dans [24] *n* = 101	Dung et al. [19] *n* = 45
Country	Singapore	Malaysia	Thailand	Philippines	Vietnam
Number of lupus nephritis (LN)	26	64	87	56	33
Number of renal biopsies (% of LN)	21 (80.7%)	44 (68.7%)	87 (100%)	24 (42.8%)	29 (87.8%)
**Histopathological classification of lupus nephritis**					
Class I	0	2.3	5.7	0	0
Class II	0	18.2	21.8	25	0
Class III	23.8	25	8	20.8	24.2
Class IV	33	36.4	56.3	50	60
Class V	4.8	4.5	8	4.1	0
Mixed III/IV + V	34.8	13.7	NA	*	3.0

* One had mixed type and classified under the predominant histology. NA: not available.

**Table 3:** Disease activity scores in childhood-onset systemic lupus erythematosus.

Disease Activity (SLEDAI) Scores	Tan et al. [21]	Lim et al. [22]	Dung et al. [19]	Hiraki et al. [11]	Watson et al. [26]
At diagnosis	16.7 (2–33)	20.1 (±9.6)	23.8 (±11.6)	13.1 (±8.4)	12
6 months	5.4 (0–33)	5.0 (±4.9)	NA	2.9 (±3.0)	NA
12 months	1.9 (0–11)	4.1 (±4.0)	NA	3.0 (±3.3)	4

NA: not available.

## Data Availability

Not applicable.
